# Stop using “former”: preterm birth and cardiometabolic risk to future health

**DOI:** 10.1038/s41390-025-04472-5

**Published:** 2025-10-07

**Authors:** Michelle M. Kelly

**Affiliations:** Villanova University, M. Louise Fitzpatrick College of Nursing, Villanova, PA USA

## Abstract

Preterm birth conveys risk to future health across the life course particularly for non-communicable conditions associated with cardiometabolic risk. The intersection of social, economic, environmental, and biobehavioral risk and preterm birth complicate the timing and expression of cardiometabolic risk. A multifaceted approach to combating risk includes providing anticipatory guidance and support to families with children born preterm that mitigate risk and promote health.

Commentary on: Cardiometabolic Characteristics of School-Aged Children Born Preterm with Very Low Birth Weight

Preterm birth is a global phenomenon with an estimated prevalence of 10% of life births, which is believed to be an underestimation of the true rate of preterm birth in low and middle-income countries due to the potential for misclassification of stillbirths versus infants born too small or too early for intervention.^1^ Outcome studies related to the health of children, adolescents, and adults who were born preterm provide an opportunity to understand the influence of early life on future health. The Developmental Origins of Health and Disease theory suggests a strong association between early life (pre-natal and post-natal periods), environment, and future states of health, with both low birth weight (birth weight less than 2.5 kg) and preterm birth (birth before the completion of 37 weeks of gestation) as key features of early life.^2^ It is critically important that healthcare teams and researchers understand the life course implications of early birth, specifically the ways early life stressors influence future adolescent and adult health. This is of particular importance related to non-communicable and multifaceted conditions like adult cardiovascular disease. Preterm birth is associated with cardiometabolic risk, even at later gestational ages,^3^ although the timing and precise mechanism of increased risk remains unclear. Much of the research focusing on cardiometabolic risk is aimed at identifying the onset of risk factors associated with cardiovascular disease such as hypertension, obesity, dyslipidemia and glucose dysregulation.^2^ Complicating the study of risk factors are the social, economic, environmental, and biobehavioral conditions that contribute to cardiovascular risk.

In this edition of *Pediatric Research*, Fernandes et al.,^4^ explore the cardiometabolic risk of school-aged children born preterm in a Brazil, a low-and middle income country, with a preterm birth rate reported to be between 10–11% annually. This study is a component of a larger study exploring the long-term physical health outcomes of children born preterm and very low birth weight (<1500 grams) between 2008 and 2012 in Rio Grande do Sul Brazil.^4^ Maternal variables, neonatal course variables, current health and socio-economic factors were examined.^4^ The sample include 77 preterm very low birth weight children and 59 full-term controls, both groups were approximately 11 years old.^4^ The preterm group was determined to have a significantly higher odds ratio for elevated blood pressure compared to full-term controls, however no significant difference in dysglycemic or dyslipidemia between the groups.^4^ Interestingly, the higher than expected rate of obesity was found in both samples, 14% in the preterm sample, 30% in the full term sample, while the reported prevalence of childhood obesity is 12% in Brazil.^4^

One known difficulty in research for people born preterm beyond the neonatal period is the ability to access a control group with adequate contemporaneously collected maternal and infant health data. As noted by Fernandes et al.,^4^ medical records for infants cared for in typical maternity wards may lack the level of maternal data needed for these explorations or be inaccessible to the researcher. The authors utilize a unique sampling strategy to overcome this challenge by selecting the full term control group from infants admitted to the neonatal intensive care unit, during the same time period for “non-serious conditions” to facilitate access to maternal and infant medical information.^4^ This strategy yielded an adequate control group sample and provided robust maternal and infant health data for comparison to the preterm sample. This is an inventive use of low acuity, full-term neonatal intensive care unit admissions which significantly increased the rigor and strength of the results.

Similar to Fernandes et al.,^4^ other researchers have attempted to pinpoint the risk and onset of non-communicable diseases like chronic kidney disease,^5^ cardiovascular risk,^6,7^, and asthma^8^ using large or national samples collected for other purposes. In a study published in 2025 by Clayton et al., multilevel models were used to examine multiple cardiometabolic risk factor trajectories from childhood to early adulthood in a sample born in the United Kingdom during the 1990s.^6^ Clayton et al. determined that preterm participants had lower lean and fat mass at age 9 compared with full-term participants as well as marginally higher systolic blood pressure from age 7 to 25 years.^6^ Kelly and Brace, using a United States sample, conducted survival analyses compared the onset of high blood pressure, cholesterol, and diabetes in 4 groups (preterm low birth weight, preterm normal birth weight, full term low birth weight, and full term normal birth weight).^7^ Kelly and Brace established a 12% earlier diabetes diagnosis for the preterm low birth weight group compared with the normal birth weight groups. Analyses of hypertension onset suggested that birth weight status was a factor but did not meet statistical significance.^7^

Preterm birth outcomes are notoriously difficult to predict, as objective measures like gestational age, birth weight, and even neonatal acuity fail to adequately explain or predict outcomes. Raju et al.,^9^ following an extensive review of adult outcomes of preterm birth survivors, put forth a conceptual model of environmental exposures from conception through delivery, childhood and into adolescence that affect the future outcomes (Fig. 1). Essential to this framework, and to the understanding of preterm birth outcomes, is the influence of familial genetic factors (maternal, paternal and grandparents), socio-economic and lifestyle factors.^9^ Socioeconomic factors dictate access to care, nutrition, availability of safe physical activity opportunities, and suggest levels of environmental stress known to contribute to both preterm delivery and poorer health outcomes. To capture this influence, Fernandes et al.^4^ examined socioeconomic factors, specifically maternal education, family income, and exposure to second-hand smoke for potential relationships with cardiovascular risk. The influence of obesity on cardiovascular health is well established in child and adult health. Fernandes et al.^4^ stress the importance of providing anticipatory guidance to families related to appropriate physical activity goals and nutritional support for children born early to limit the incidence of obesity and the subsequent additional risk to cardiometabolic health.

**Fig. 1:**
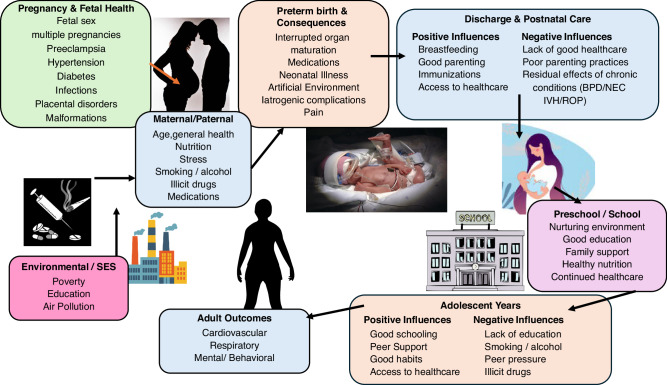
Life-course perspective & conceptual framework for pathways that may affect long-term outcomes in adults born preterm. Used with permission form Satyan Lakshminrusimha, MD & Tonse Raju MD. Originally published in: Raju TNK, Buist AS, Blaisdell CJ, Moxey-Mims M, Saigal S. Adults born preterm: a review of general health and system-specific outcomes. *Acta Paediatr*. 2017;106(9):1409–1437. 10.1111/apa.13880.

This research, and other studies like it, contribute to the understanding of the outcomes of children, adolescents, and adults who survive and thrive after being born early. In the United States and in Brazil, the preterm birth rate has remained stable around 10–11% for decades. Two-thirds of preterm births occur without a currently identifiable biologic etiology worldwide,^10^ which means that the epidemic of preterm birth is not going away. Based on the stable preterm birth rates and high survival rates, it is hypothesized that every healthcare provider, regardless of practice specialty or population, cares for people who were born preterm, most without knowing this vital piece of past medical history. Preterm birth history increases an individuals risk of non-communicable diseases with the proportion of risk related to gestational age and birth weight, but remaining present in those born at later gestations and larger birth weights.^3,7,9^

It is time for preterm birth to become a standard component of past medical history. People born preterm are not “former 30-weekers” as the effects of preterm birth are not left at the door of the neonatal intensive care unit. Rather people born early are a unique population with an established risk to future health, that behooves clinicians to document gestational age and birth weight in electronic medical records and understand the gradient of risks that are conferred. The proliferation of credible research findings over the last 10–20 years, support the use preterm birth in risk based decision making, much the way personal history of smoking or family history of cardiovascular disease or cancer are used. This is a call for faculty and practicing clinicians to impart this knowledge to students and clinician trainees. Further, there is an urgent need to translate the preterm birth outcome findings into actionable clinical care and anticipatory guidance to establish the promotion of health and mitigation of risk for people born preterm.
